# Positive memory increases cataplexy-like behaviors in narcolepsy mice as revealed using conditioned place preference test

**DOI:** 10.1186/s12868-022-00772-2

**Published:** 2022-12-28

**Authors:** Mayuko Yoshida, Koki Yamamoto, Tomoyuki Kuwaki

**Affiliations:** Department of Physiology, Graduate School of Medical and Dental Sciences, Kagoshima University, Sakuragaoka 8-35-1, Kagoshima, 890-8544 Japan

**Keywords:** Positive emotion, Cataplexy behavior, Nucleus accumbens, Orexin neurons, Extracellular signal-regulated kinase

## Abstract

**Background:**

Cataplexy is a loss of muscle tone that can lead to postural collapse, disturbing the daily life of narcolepsy patients; it is often triggered by positive emotions such as laughter in human patients. Narcolepsy model mice also show cataplexy, and its incidence increases in response to positive emotion-inducing stimuli such as chocolate and female courtship. Although such observation indicates a positive emotion-related nature of cataplexy in narcolepsy mice, they also show cataplexy without any apparent triggering stimulus ~ (spontaneous cataplexy). Therefore, we hypothesized that some spontaneous cataplexy in narcoleptic mice might indicate the remembering of happy moments.

**Results:**

To test our hypothesis, we did a conditioned place preference test on orexin/hypocretin neuron-ablated (ORX-AB) mice, one of the animal models of human narcolepsy, and counted the number of cataplexy-like behaviors. ORX-AB mice successfully remembered the chocolate-associated chamber, and the number of cataplexy-like behaviors significantly increased in the chocolate-associated chamber but not in the control chamber. In addition, ORX-AB mice remembered the aversive odor-associated chamber and avoided entering without affecting the number of cataplexy-like behaviors. Finally, similar activation of the nucleus accumbens, a positive emotion-related nucleus, was observed during both spontaneous and chocolate-induced cataplexy behaviors.

**Conclusions:**

These results support our hypothesis and will promote the usefulness of a narcolepsy mice model in emotion research and serve as a basis for a better understanding of cataplexy in narcolepsy patients.

**Supplementary Information:**

The online version contains supplementary material available at 10.1186/s12868-022-00772-2.

## Introduction

Cataplexy is a loss of muscle tone that can lead to postural collapse, disturbing the daily life of narcolepsy patients. It is a primary symptom of narcolepsy, caused by the abnormal loss of orexin (hypocretin)-producing neurons in humans [1], orexin deficiency in mice [2, 3], and orexin receptor mutation in dogs [4]. Cataplexy is triggered by positive emotions, such as laughter, in human patients [5, 6]. Narcolepsy model mice also show cataplexy, and its frequency increases in response to positive emotion-inducing stimuli such as chocolate and female courtship [7–9]. Chocolate increased the incidence of cataplexy by ~ 60% compared to regular food in orexin neuron-ablated mice [9]. Although this observation indicates the positive emotion-related nature of cataplexy in mice with narcolepsy, it also shows cataplexy without any apparent triggering stimulus [2, 9]. We refer to this as spontaneous cataplexy in this paper. The mechanism for spontaneous cataplexy has not been revealed yet.

In nocturnal mice, both spontaneous and emotionally-induced cataplexies are mainly observed during the active dark period [2, 8–11]. An animal model lacking both orexin neurons and melanin-concentrating hormone (MCH) neurons loses circadian regulation of cataplexy [12], indicating the inhibitory role of MCH on daytime cataplexy. At least for chocolate-induced cataplexy, activation of the prefrontal cortex and the nucleus accumbens (NAc) is thought to be an upstream trigger [7, 8] of cataplexy.

Among human narcolepsy patients, of those who were proven to be orexin deficient, about 60% reported that they sometimes experienced spontaneous cataplexy in addition to laughter-induced cataplexy [5]. In addition, patients reported that they sometimes experienced cataplexy with no particular trigger, such as while laughing, joking, feeling angry, feeling stressed, eating, feeling pain, etc., based on the multiple-choice questionnaire that included a wide range of emotions/situations (at least 24 choices) [5]. Another report showed that remembering a happy moment was one of the triggers of cataplexy [6].

Based on this research, we hypothesized that some spontaneous cataplexy in narcoleptic mice might indicate the remembering of happy moments. To test our hypothesis, we used a combination of the conditioned place preference/avoidance test and counting cataplexy-like behaviors in the test chamber. We also compared the magnitude of activation in the NAc between spontaneous cataplexy and chocolate-induced cataplexy.

## Results

**Fig. 1:**
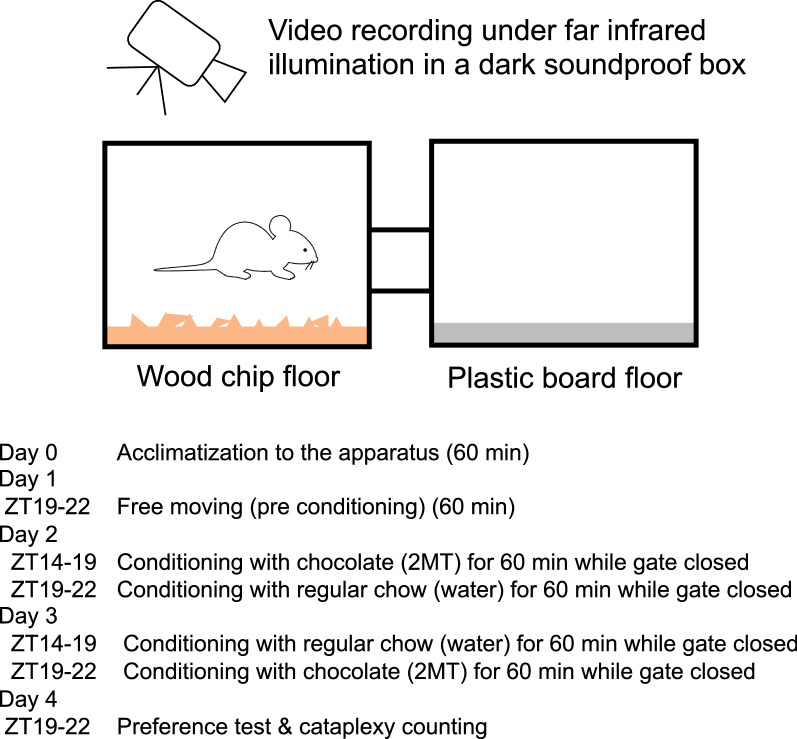
Experimental procedure. The place preference test was performed in a dark environment. To make a clear contrast between the test chambers, we selected two different floor materials: wood chips and plastic boards. Test chambers were connected with a tube with a gate kept open during pre-conditioning measurement on day 1 and the test period on day 4. On day 2 and day 3, mice received a piece of chocolate in one chamber, and regular chow in the other with the gate closed. Another set of animals was conditioned using an instinctively aversive odorant, 2-methyl-2-thiazoline (2MT), and distilled water as the control. The method section contains a detailed description

**Fig. 2:**
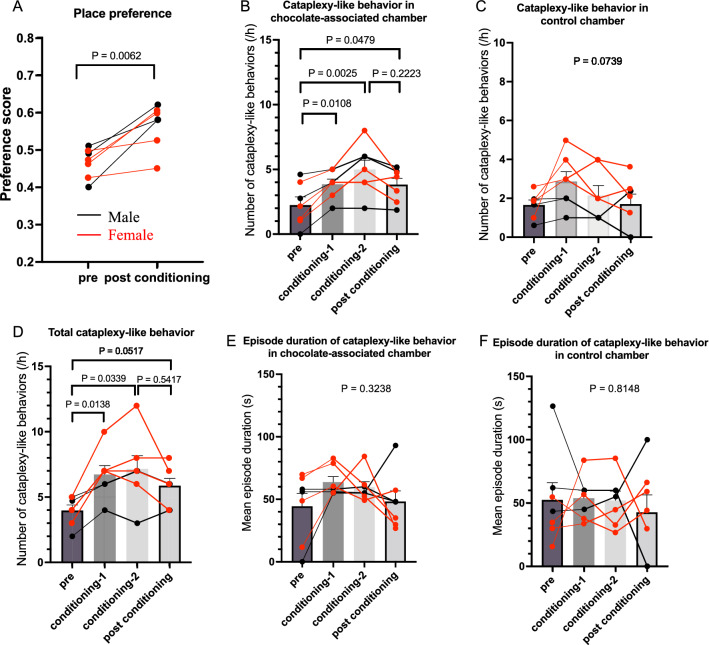
Positive conditioning increases the number of cataplexy-like behaviors. For this study, we used three male (black dots and lines) and four female (red dots and lines) orexin neuron ablated mice. **A** The preference score was calculated by dividing the time spent in the treatment-paired chamber by the total time spent in both chambers. None of the animals had an initial bias (preference score > 0.8 or < 0.2) for either chamber. Two days of conditioning with chocolate successfully increased the preference score (2-way ANOVA). **B**-**D** The number of cataplexy-like behavior in the chocolate-associated chamber **B**, in the control chamber **C**, and total number **D** are shown. Bars indicate mean ± SEM of the mixed data from male and female mice. The number of cataplexy-like behaviors during pre- and post-conditioning periods was normalized by the stay time in the chamber. There was no need for such data normalization during conditioning-1 and conditioning-2 because the mice were confined to one chamber during the conditioning period. In the chocolate-associated chamber, cataplexy-like behavior significantly increased during the post-conditioning period compared to the pre-conditioning period. To examine whether there was a difference in the characteristics of cataplexy-like behavior among pre-conditioning, conditioning, and post-conditioning periods, we also calculated the duration of a single cataplexy-like episode. It did not change when the animal stayed in the chocolate-associated chamber **E** and the control chamber **F**. Together, positive conditioning seemed to affect the starting but not maintenance of cataplexy-like behavior. Statistical significances denoted in the figure were obtained by 2-way ANOVA in (A) and by repeated design one-way ANOVA followed by Sidak's multiple comparison test, if appropriate, in (**B**–**F**)

**Fig. 3:**
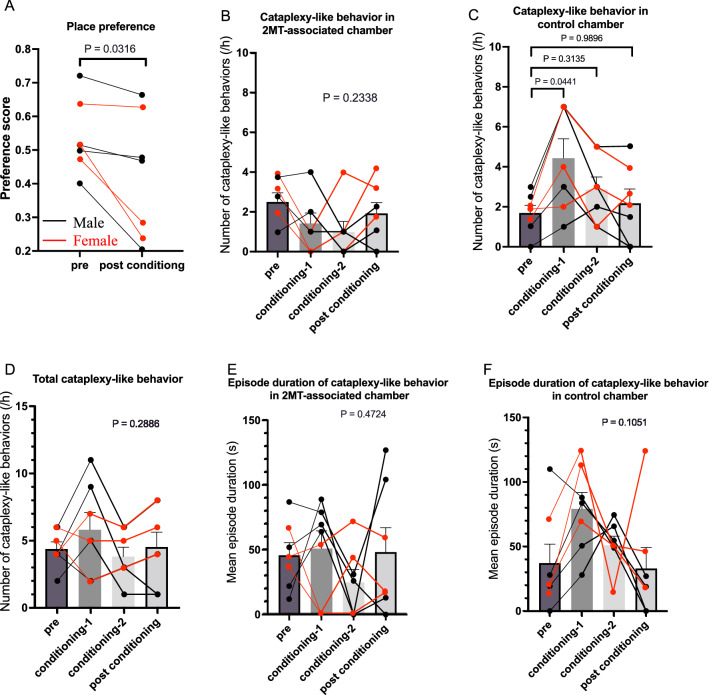
Negative conditioning did not change the number of cataplexy-like behavior. We used a different set of naïve orexin neuron ablated mice (four males and three females) in this study. **A** Two days of conditioning with an instinctively aversive odorant, 2-methyl-2-thiazoline (2MT), induced avoidance behavior, as shown by a significant decrease in preference for the 2MT-associated chamber. Meanwhile, the number of cataplexy-like behavior **B**–**D** and the duration of a single cataplexy-like episode **E**, **F** did not change by negative conditioning, except for a significant increase in the control chamber during conditioning-1 (**C**). Statistical significances denoted in the figure were obtained by 2-way ANOVA in (A) and by repeated design one-way ANOVA followed by Sidak's multiple comparison test, if appropriate, in (**B**–**F**)

We first confirmed that the conditioned place preference/avoidance test could be executed effectively in a dark environment (Fig. 1). None of the animals had an initial bias (preference score > 0.8 or < 0.2) for either chamber. Touch sensation from floor texture in the darkroom was a sufficiently effective cue to discriminate the chocolate-associated chamber from the regular chow-associated chamber (Fig. 2A). Preference score significantly increased from 0.466 ± 0.001 (mean ± SEM) to 0.569 ± 0.024 by chocolate conditioning (F_1,5_ = 20.63, P = 0.0062, repeated measures 2-way (test day and gender) ANOVA, Supplemental Fig. 1). A different set of animals successfully discriminated an aversive odor-associated chamber from odorless water vapor-associated chamber (Fig. 3A). Preference score significantly decreased from 0.537 ± 0.003 to 0.418 ± 0.036 by aversive odor conditioning (F_1,5_ = 8.748, P = 0.0316). There was no apparent difference between male and female mice in the chocolate preference test (F_1,5_ = 0.5918, P = 0.4765) and the odor aversion test (F_1,5_ = 0.0785, P = 0.7906). Therefore, cataplexy-related scores (number and duration) described below were treated as a whole for both sexes.

In the pre-conditioning period, the number of cataplexy-like behaviors in the wooden tip floor chamber (2.2 ± 0.3 times/h, mean ± SEM, n = 14) was not statistically different from that in the plastic floor chamber (1.9 ± 0.3, P = 0.541, unpaired t-test), indicating the efficiency of the current apparatus to study conditioned place preference and cataplexy-like behavior simultaneously.

Mice showed significantly more cataplexy-like behaviors when chocolate was present (Fig. 2B), from 2.25 ± 0.63 bouts/h during the pre-conditioning period to 3.86 ± 0.40 bouts/h during the 1st conditioning period (P = 0.0108, repeated measure one-way ANOVA and Sidak’s multiple comparisons test) and to 5.00 ± 0.72 bouts/h during the 2nd conditioning period (P = 0.0025). During post-conditioning testing, mice still showed a significantly increased number of cataplexy-like behaviors (3.84 ± 0.49 bouts/h, P = 0.0479) even though no chocolate was available. Although the number of cataplexy-like behaviors was lower than during the 2nd conditioning period, there was no statistically significant difference between the two (3.84 vs. 5.00, P = 0.2223). Meanwhile, the number of cataplexy-like behaviors in the control chamber did not significantly change during the conditioning periods or during the post-conditioning testing period (P = 0.0739, repeated measure one-way ANOVA) (Fig. 2C). The total number of cataplexy-like behaviors significantly increased during the conditioning periods. In addition, it tended to increase during the post-conditioning testing period (Fig. 2D). Increases seemed mainly attributable to the increase in the number of cataplexy-like behaviors in the chocolate-associated chamber. Note that the number of cataplexy-like behaviors in Fig. 2B, C was normalized by the stay time in the chamber to avoid overestimation by the high stay time in the chocolate-associated chamber. In contrast to the apparent difference in the number of cataplexy-like behaviors, the duration of a single cataplexy-like episode was not affected by the chocolate conditioning in both the chocolate-associated chamber (Fig. 2E) and the control chamber (Fig. 2F).

In the conditioning sessions, mice consumed more chocolate (0.41 ± 0.04 g, n = 14) than the regular chow (0.26 ± 0.03 g, P = 0.002, paired t-test), indicating positive attraction of the chocolate. However, there was no significant difference in the consumed amount of chocolate between the conditioning day-1 (0.41 ± 0.05 g, n = 7) and day-2 (0.43 ± 0.06 g, P = 0.736, paired t-test). Furthermore, within the conditioning sessions, mice showed a significantly larger number of cataplexy-like behaviors in the chocolate-fed room (4.4 ± 0.4 times/h, mean ± SEM, n = 14) than in the regular chow room (2.5 ± 0.4, P = 0.003, paired t-test), indicating that chocolate induced cataplexy-like behavior in this experimental setup.

Mice showed significant avoidance of an aversive odorant, 2-methyl-2-thiazoline (2MT)-associated chamber (Fig. 3A). Mice’s cataplexy-like behavior tended to decrease in the chamber with 2MT (Fig. 3B) during the conditioning periods. However, there was no significant difference among the periods, including the post-conditioning testing period. In the control chamber, mice showed significantly more cataplexy-like behaviors during the 1st conditioning period (Fig. 3C), from 1.69 ± 0.38 bouts/h during the pre-conditioning period to 4.43 ± 0.97 bouts/h during the 1st conditioning period (n = 7, P = 0.0223, repeated measure one-way ANOVA and Sidak’s multiple comparisons test). Nevertheless, the number of cataplexy-like behaviors was not different between pre- and post-conditioning (2.17 ± 0.71 bouts/h, P = 0.898) periods. In addition, the total number of cataplexy-like behaviors did not significantly change with 2MT-conditioning (Fig. 3D). Duration of cataplexy-like episodes (Fig. 3E, F) did not significantly change throughout all the periods. The results indicate that aversive memory recall has no constant effect on cataplexy-like behavior, even if the presence of an aversive stimulus may have a slight inhibitory effect.

**Fig. 4:**
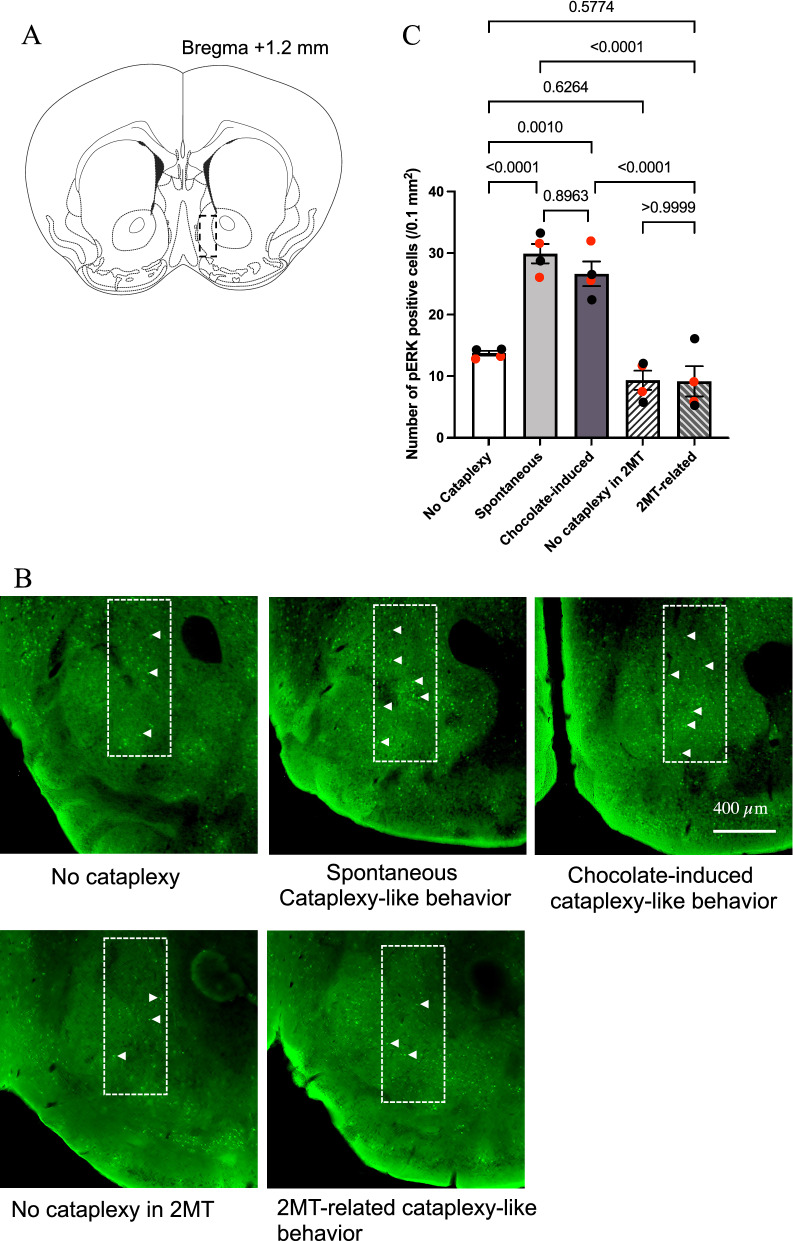
Similar activation of the NAc during both spontaneous and chocolate-induced cataplexy-like behavior. Coronal brain slices, including the nucleus accumbens (NAc), **A** were obtained from five groups of mice. No cataplexy group (n = 4); mice were anesthetized after 10 min of the cataplexy-free period, and the brain was sampled. Spontaneous cataplexy-like behavior group (n = 4); when the mice showed cataplexy-like behavior without chocolate and any other apparent stimulus, they were immediately anesthetized, and the brain was sampled. Chocolate-induced cataplexy group (n = 4); when the mice showed cataplexy-like behavior within 1 min after the chocolate bite, they were immediately anesthetized, and the brain was sampled. In the no cataplexy in the 2MT group (n = 4), mice were anesthetized after 10 min of a cataplexy-free period in a cage with 2MT, and the brain was sampled. In the 2MT-related cataplexy-like behavior group (n = 4), when the mice showed cataplexy-like behavior in a cage with 2MT, they were immediately anesthetized, and the brain was sampled. Photographs in **B** show typical examples from five groups of the brain. According to our previous method [7], the number of pERK-positive cells was counted in the area indicated by a dashed rectangle (400 × 1000 µm) in the NAc shell. **C** Statistical results using Sidak's multiple comparison test showed significantly higher numbers of pERK-positive cells in the chocolate-induced and spontaneous cataplexy group than in other groups. There was no significant difference between chocolate-induced and spontaneous cataplexy groups. Each group consists of two females (red dots) and two males (black dots)

Narcolepsy model mice show cataplexy behavior, even without apparent positive emotion-inducing stimulus [9]. To test if such spontaneous cataplexy is similar to chocolate-induced cataplexy, we next examined the activation of the nucleus accumbens (NAc), a putative trigger site for chocolate-induced cataplexy [7], using a phosphorylated form of the extracellular signal-regulated kinase (pERK) as a marker of cellular activation [7]. We used 20 (10 male and 10 female) ORX-AB mice for this purpose and compared the number of pERK-positive cells in the NAc among the no-cataplexy, spontaneous cataplexy-like behavior, chocolate-induced cataplexy-like behavior, no-cataplexy in the cage with 2MT, and cataplexy-like behavior in the cage with 2MT groups (Fig. 4). There was a significant difference in the number of pERK positive cells among the five groups (F_4, 15_ = 32.05, P < 0.0001, one-way ANOVA). As expected, the brains from the chocolate-induced cataplexy-like behavior group (26.6 ± 2.0/0.1 mm^2^, mean ± SEM, n = 4) showed significantly larger numbers of pERK positive cells (P = 0.0010, Sidak’s multiple comparison test) than those from the no-cataplexy group (13.7 ± 0.4, n = 4) (Fig. 4C). The brains from the spontaneous cataplexy-like behavior group (29.9 ± 1.6, n = 4) also showed significantly larger numbers of pERK positive cells (P < 0.0001) than those from the no-cataplexy group but did not show a difference from those in the chocolate-induced cataplexy-like behavior group. There was no significant effect of 2MT by itself on the number of pERK, as evidenced by the lack of difference between the no-cataplexy group and the no-cataplexy in the 2MT group. The number of pERK in the 2MT-related cataplexy-like behavior group was significantly smaller than that in the chocolate-induced cataplexy-like behavior group and that in the spontaneous cataplexy-like behavior group, indicating positive emotion-related nature rather than cataplexy-related nature of pERK expression in the NAc. These results showed a similarity between chocolate-induced and spontaneous cataplexy-like behaviors and a difference between those two and 2MT-related cataplexy-like behavior.

## Discussion

We hypothesize that orexin neuron-ablated (ORX-AB) mice, one of the models of human narcolepsy, sometimes recall positive emotion-inducing experiences without apparent stimulus. Such positive emotion triggers spontaneous cataplexy-like behavior in them. To test our hypothesis, we used a combination of conditioned place preference/avoidance tests and counting cataplexy-like behaviors in the test chamber. We first examined whether ORX-AB mice remember positive and negative conditioning during the dark period. The preference score increased with chocolate and decreased with 2MT odor (Figs. 2A, 3A). This result is in accordance with the previous report [13] that ORX knockout mice could not work for positive reward during the light phase but not during the dark phase or when working to avoid shock in the light or dark phase. It should be noted here that ORX-AB mice could perform preference and avoidance learning even though the activity of orexin neurons is somewhat related to preference learning [14].

ORX-AB mice showed increased cataplexy-like behaviors during chocolate conditioning but not during 2MT conditioning. After 2 days of conditioning, ORX-AB mice showed an increased number of cataplexy-like behaviors in the chocolate-associated chamber but not in the control chamber or the aversive odor-associated chamber, as compared to pre-conditioning values. Since there was no apparent trigger to induce emotional change during the test period, a change in cataplexy-like incidence should be attributed to memory. We also found similar activation of the NAc, one of the trigger sites for cataplexy [7], in spontaneous cataplexy-like behavior and chocolate-induced cataplexy-like behavior groups but not in 2MT-related cataplexy-like behavior group (Fig. 4). We propose that recalling positive memory increases the incidence of cataplexic attack through a brain mechanism similar to that of mice in the presence of a positive emotion-inducing stimulus. In this study, positive memory increased the number of cataplexy-like behaviors. However, it did not affect the duration of a single cataplexy-like episode (Fig. 2). Courtship-associated [9] and food-associated [15] increases in cataplexy are characterized mainly by an increase in frequency but to a lesser extent by the duration of an episode. Such similarity also indicated a similar brain mechanism. Nevertheless, not all spontaneous cataplexy seemed to be related to positive memory recall since a similar number of cataplexy-like behaviors was observed in the control chamber after aversive conditioning as during the pre-conditioning period (Fig. 3). It is interesting to note that cataplexy-like behavior in the control chamber significantly increased on the first day of conditioning (Fig. 3C). Although there was no reward or punishment in the control chamber, relief from the aversive chamber may have induced positive emotion. Mice seem to recall happy moments and feel relief without actual triggers in front of them, just as humans do.

Recent studies showed a gender difference in the number and duration of cataplexy in orexin neuron-ablated mice [16, 17] and orexin peptide knockout mice [18]. However, we did not find any gender difference not only in the place preference score (Fig. 2A and result section) but also in the cataplexy-like behavior in the chocolate-associated chamber (Additional file: 1 Fig. 1S). We do not know the reason for this apparent discrepancy, but the hedonic effect of chocolate may be strong enough to override the gender difference.

## Limitations

We have not measured EEG/EMG in this study and judged cataplexy-like behavior by video recordings. We admit this method is convenient but error-prone, especially when another type of behavioral arrest, such as sleep and freezing, is expected. However, mice did not stay in one place for more than 4 min in our experimental setup, indicating that they spent most of their time awake. In the test of the aversive effect of 2MT, our data might be overcounting since this compound is known to induce freezing behavior [19]. However, our data showing a tendency to reduce cataplexy-like behavior in the 2MT-associated chamber (Fig. 3B) did not support false counting of freezing as cataplexy.

Immunohistochemistry with pERK has better time resolution (a few minutes) than that with c-Fos (hours) [20] and can be used to detect cataplexy-related cellular excitation [7]. Nevertheless, since a cataplexy attack suddenly happens and lasts from a few seconds to minutes, more precise methods, such as fiber photometry [21], is needed in future study.

The conditioned place preference/avoidance test is one of the best behavioral measures to estimate the animal's emotions and memory. However, we can only speculate, not determine, the animals' emotions and memory by their behavior since we cannot verbally communicate with them. Therefore, it is interesting to examine whether spontaneous cataplexy in human narcolepsy patients is associated with the memory of happy moments. When proven, knowledge from animal studies can be convincingly applied to better treatment of human narcolepsy, and development will be accelerated.

## Conclusions

Here, we described the positive-memory associated increase of cataplexy-like incidence in narcolepsy mice. Spontaneous cataplexy-like behavior seems to occur through a similar brain mechanism as positive emotion-induced cataplexy-like behavior. This notion will promote the usefulness of a narcolepsy mice model in emotion research and serve as a basis for a better understanding of cataplexy in narcolepsy patients.

## Materials and methods

### Ethics approval

All experiments were conducted at Kagoshima University following the guiding principles for the care and use of animals in physiological sciences published by the Physiological Society of Japan (2015) and were approved by the Experimental Animal Research Committee of Kagoshima University (MD17105).

### Animals

For the animal model of narcolepsy, 17 male and 17 female orexin neuron-ablated (ORX-AB) mice [7, 9, 10] were used in a balanced number of sexes for each experiment. They were 8–16 weeks old at the start of the experiment and weighed 22–32 g. The original mating pairs were a generous gift from Prof. Yamanaka at Nagoya University and bred in Kagoshima University’s animal facility. A method for selective ablation of orexin neurons has been previously reported [10]. Essentially, orexin-tTA mice that express tetracycline transactivator (tTA) exclusively in orexin neurons under the control of the human prepro-orexin promoter [10] were bred with tetO diphtheria toxin A fragment (DTA) mice (B6.Cg-Tg (tetO DTA) 1Gfi/J, The Jackson Laboratory) to generate orexin-tTA; tetO DTA mice. In these double-transgenic mice (called ORX-AB in this paper), doxycycline is removed from their chow starting from birth so that by four weeks of age, almost all (> 97%) of orexin neurons are ablated [10]. Our previous studies confirmed the ablation of orexin neurons [7, 9, 22]. All mice were housed in a room maintained at 22–24 °C with lights on at 19:00 and off at 7:00 at least two weeks before experimentation started. We selected a reversed light/dark cycle so experimenters could observe mice in their active nocturnal phase of behavior during the daytime. Mice had food and water available ad libitum. The mice were grown in a group before the experiment. During the experimental period, they were singly housed to avoid any possible social rank effect on behavior [23].

### Behavioral observation of cataplexy

A far-infrared lamp illuminated the experimental chamber in a light-controlled soundproof box (940 nm, SA2-IR, World Musen, Hong Kong). Mouse behavior was continuously recorded with a video camera (CBK21AF04, Imaging Source Asia, Taipei, Taiwan) and monitored on a computer outside the soundproof box. Cataplexy was determined according to the established criteria for mice [24], defined by several observable features. The first feature is an abrupt episode of nuchal atonia lasting at least 10 s. Atonia was determined to occur when mice were prone with their head and belly down in the bedding with their limbs and tail typically situated straight out from the trunk. This posture contrasts with a normal sleeping position in which mice are curled up and fold their limbs and tail underneath their trunk. Second, the mouse is immobile aside from the movements associated with breathing during an episode. Finally, at least 40 s of active wakefulness (moving) preceding the atonia episode. The original criteria recommend recordings of EEG, but we did not adopt EEG to avoid possible obstruction of mice movement by the recording cable. Therefore, we use “cataplexy-like behavior” instead of “cataplexy” when describing current results in this manuscript.

### Conditioned place preference test

We used a homemade place preference apparatus to examine the possible effect of memory on the number of cataplexies (Fig. 1). Two plastic chambers (14 × 20 × 15 cm) were connected via a tube that allowed the animal to move back and forth freely. The floor of one chamber was filled with wood chip bedding and the other with a plastic board, so there was a clear difference between the two. Since our test was performed during the dark period, visual cues by wall color would not be as effective as a popular conditioned place preference test that uses both visual and tactile cues by floor texture for memory recall. The test chambers were thoroughly cleaned after each test to avoid any odorous cues.

The mice were first allowed to explore the apparatus for acclimatization for 60 min freely. The next day, we tested whether the mice preferred a specific side chamber or a specific floor material while both chambers were empty, except for floor material, for one hour. The mice's position was judged by the tip of the nose and assigned to one chamber, even if the part of the body was in the connecting tube. The time spent in each chamber was recorded to assess the basal preference (pre-value). The preference score was calculated by dividing the time spent in the treatment-paired chamber by the total time spent in both chambers. Conditioning sessions were conducted for two consecutive days. In the first conditioning session at ZT14-17 for mice, mice were confined to one chamber for 60 min with a piece of milk chocolate (Hershey’s Kiss; Hershey). At ZT19-22 for mice, we confined the mice to the other chamber with a regular chow. During the two conditioning sessions, mice were returned to their home cage in the dark room. On the second day, the first session was a regular chow treatment, and the second was a chocolate treatment. A combination of floor texture and treatment was randomly assigned to the mice. On the day after the last conditioning session, the preference test was conducted in the same manner as the basal preference assessment at ZT19-22. Throughout all sessions, cataplexy behavior was blindly detected from video recordings to the treatment.

To examine the possible effect of aversive memory on cataplexy-like behavior, we performed a similar conditioned place preference (avoidance) test using an instinctively aversive odorant, 2-methyl-2-thiazoline (2MT; 2346-00-1, Henan Alfa Chemical, Qinyang City, China) [19], in place of chocolate described above. In this experiment, 0.5 ml of 2MT in a glass bottle (50 ml) that has a metal lid with small holes was placed in the chamber. The control was distilled water. To avoid a possible carry-over effect, we used a different set of naïve animals for this experiment.

After each observation, place preference apparatus cages were thoroughly washed with cleaner, sterilized with hypochlorous acid, and air dried. Observation using male and female mice was performed on different days to avoid possible cross-over effects from smell and sound.

### Immunohistochemistry

To examine whether spontaneous cataplexy activates the NAc as chocolate-induced cataplexy did [7], we measured a cellular activation marker, pERK. We selected to measure pERK but not c-Fos since pERK is a cellular activation marker with a more rapid and narrow time window than other activation markers [20]. Our previous study examined brain regions activated at the initiation of chocolate-induced cataplexy. We found that the rostral part of the NAc was the only region out of 33 brain regions examined when pERK was used as an activation marker [7]. Therefore, we focused on NAc in this study. We examined five groups of mice brains that were sampled at ZT14-17. (1) Chocolate-induced cataplexy-like behavior group: When the mice showed cataplexy-like behavior within one min after the chocolate bite, they were immediately euthanized with a lethal dose of urethane (1.8 g/kg, i.p.) and bleeding, transcardially perfused with phosphate-buffered saline (PBS, 0.01 M, pH 7.4), followed by 4% paraformaldehyde (PFA) in PBS and the brain was removed. (2) Spontaneous cataplexy group: When the mice showed cataplexy-like behavior without chocolate in his/her home cage, their brain was sampled as described above. (3) The control group: The mice were euthanized after confirmation of no cataplexy for 10 min. (4) 2MT-related cataplexy-like behavior group: We did not define 2MT- “induced” cataplexy because mice did not show any cataplexy-like behavior within one minute after smelling the 2MT bottle, while they had when smelling and biting chocolate. Nevertheless, mice sometimes showed cataplexy-like behavior in the cage where the bottle of 2MT was settled. Therefore, we call such cataplexy-like behavior 2MT- “related.” When the mice showed cataplexy-like behavior, their brain was sampled as described above. (5) No cataplexy in the 2MT group: To examine the possible effect of 2MT alone on NAc activity, the mice were euthanized after no cataplexy occurred for 10 min in the cage with 2MT. In all five cases, the mice's behavior in the home cage was recorded by a video camera in a soundproof box and monitored on a computer screen outside the soundproof box [7]. The experimenter manually judged cataplexy-like behavior, and the brain was sampled within approximately five min after injection of the anesthetic. The brains were post-fixed in 4% PFA solution at 4 °C overnight and immersed in 30% sucrose in PBS at 4 °C for two days. Coronal sections, including the NAc, were cut at 40 µm thickness using a vibratome, and every fourth Section (10 slices from each specimen) was used for immunostaining. The brain sections were immersed in a blocking solution (1% normal horse serum and 0.3% Triton-X in 0.01 M PBS) for 1 h at room temperature. The sections were then incubated with anti-pERK rabbit antibody (4370, Cell Signaling Technology, RRID: AB_2315112) at 1/400 diluted in blocking solution overnight. The sections were washed with PBS and then incubated with CF488-labelled donkey anti-rabbit IgG (1:500, 20015, Biotium, Heyward, CA, USA, RRID: AB_10559669) for 90 min. The sections were then mounted on a glass slide and examined using a fluorescence microscope (BZ-X700, Keyence Corp., Osaka, Japan). Among the 10 slices from each specimen, the most representative slice near the rostral NAc (1.2 mm anterior to bregma) (Fig. 4A) was selected and photographed using the same exposure time for all the slices from 5 experimental groups. The number of pERK-positive cells in the rectangle (400 × 1000 µm) in the NAc was bilaterally counted (Fig. 4B) with the assistance of the counting function in Photoshop software in a blinded manner to the treatment.

### Statistical analysis

The number of cataplexy-like behaviors during pre- and post-conditioning periods was normalized by the stay time in the chamber. There was no need for such data normalization during conditioning-1 and conditioning-2 because the mice were confined to one chamber during the conditioning period. Statistical analyses were performed using Prism software v.9 (GraphPad). Two-way (test day x gender) ANOVA was used to compare the preference score between pre- and post-values in the conditioning test. Repeated measure design one-way ANOVA with the Geisser-Greenhouse correction, followed by Sidak’s multiple comparison test, was used to compare cataplexy-related scores. One-way ANOVA followed by Sidak’s multiple comparison test was used to examine the number of pERK-positive cells. P < 0.05 was considered statistically significant. Data are presented as mean ± SEM.

## Supplementary Information

**Additional file 1**. Supplemental Table 1: Raw data obtained in this study to calculate summary statistics in the article.

**Additional file 2**. Supplemental Fig. 1: 2way (test day and gender) repeated measure ANOVA showed no difference in gender.  

## Data Availability

Summary statistics are available in the article. In addition, the Additional file 2 supporting this study's findings are available in the supporting file.

## References

[1] Thannickal TC (2000). Reduced number of hypocretin neurons in human narcolepsy. Neuron.

[2] Chemelli RM (1999). Narcolepsy in orexin knockout mice: Molecular genetics of sleep regulation. Cell.

[3] Mieda M (2004). Orexin peptides prevent cataplexy and improve wakefulness in an orexin neuron-ablated model of narcolepsy in mice. Proc Natl Acad Sci USA.

[4] Lin L (1999). The sleep disorder canine narcolepsy is caused by a mutation in the hypocretin (orexin) receptor 2 gene. Cell.

[5] Overeem S (2011). The clinical features of cataplexy: A questionnaire study in narcolepsy patients with and without hypocretin-1 deficiency. Sleep Med.

[6] Krahn LE (2005). Characterizing the emotions that trigger cataplexy. J Neuropsychi Clin Neurosci.

[7] Su J (2020). Involvement of the nucleus accumbens in chocolate-induced cataplexy. Sci Rep.

[8] Oishi Y (2013). Role of the medial prefrontal cortex in cataplexy. J Neurosci.

[9] Kuwaki T, Kanno K (2021). Sexual excitation induces courtship ultrasonic vocalizations and cataplexy-like behavior in orexin neuron-ablated male mice. Comm Biol.

[10] Tabuchi S (2014). Conditional ablation of orexin/hypocretin neurons: a new mouse model for the study of narcolepsy and orexin system function. J Neurosci.

[11] España RA (2007). Running promotes wakefulness and increases cataplexy in orexin knockout mice. Sleep.

[12] Hung CJ (2020). Dual orexin and much neuron-ablated mice display severe sleep attacks and cataplexy. eLife.

[13] McGregor R (2011). Highly specific role of hypocretin (orexin) neurons: Differential activation as a function of diurnal phase, operant reinforcement versus operant avoidance and light level. J Neurosci.

[14] Garau C, Blomeley C, Burdakov D (2020). Orexin neurons and inhibitory agrp→orexin circuits guide spatial exploration in mice. J Physiol.

[15] Clark EL (2009). Feeding-elicited cataplexy in orexin knockout mice. Neurosci.

[16] Coffey AA (2021). The impacts of age and sex in a mouse model of childhood narcolepsy. Font Neurosci.

[17] Piilgaard L (2022). Sex-related differences within sleep-wake dynamics, cataplexy, and EEG fast-delta power in a narcolepsy mouse model. Sleep.

[18] Arthaud S (2022). Effects of sex and estrous cycle on sleep and cataplexy in narcoleptic mice. Sleep.

[19] Wang Y (2018). Large-scale forward genetics screening identifies trpa1 as a chemosensor for predator odor-evoked innate fear behaviors. Nat Com.

[20] Antoine B, Serge L, Jocelyne C (2014). Comparative dynamics of mapk/erk signalling components and immediate early genes in the hippocampus and amygdala following contextual fear conditioning and retrieval. Brain Struct Funct.

[21] Zhou S (2022). Activity of putative orexin neurons during cataplexy. Mol Brain.

[22] Futatsuki T (2018). Involvement of orexin neurons in fasting- and central adenosineinduced hypothermia. Sci Rep.

[23] Shansky RM (2019). Are hormones a “female problem” for animal research?. Science.

[24] Scammell TE (2009). A consensus definition of cataplexy in mouse models of narcolepsy. Sleep.

