# Supplementary material for: Positive memory increases cataplexy-like behaviors in narcolepsy mice as revealed using conditioned place preference test
Source: BMC Neurosci. 2022 Dec 28;23:82. doi: 10.1186/s12868-022-00772-2 (PMC9798626; doi:10.1186/s12868-022-00772-2)
Supplement: Supplementary file 1 — Additional file 1. Supplemental Table 1: Raw data obtained in this study to calculate summary statistics in the article. [file 12868_2022_772_MOESM1_ESM.pdf]

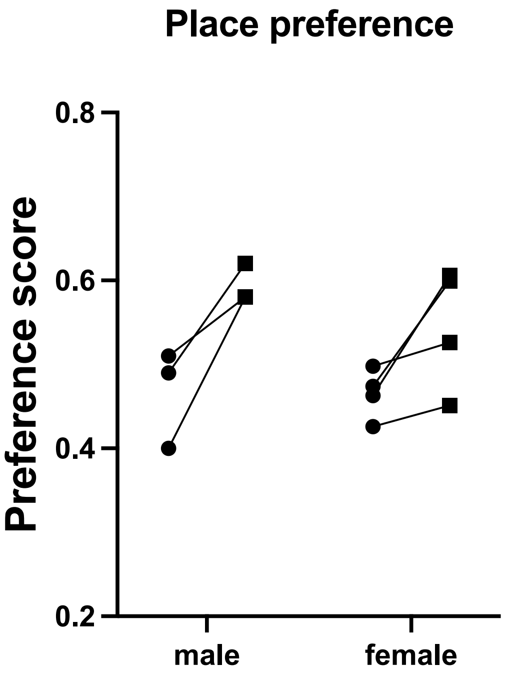

2way RM ANOVA results

Test day x gender	F(1,5) = 1.038, P = 0.355
Gender	F(1,5) = 0.5918, P = 0.4765
Test day	F(1,5) =20.63, P = 0.0062

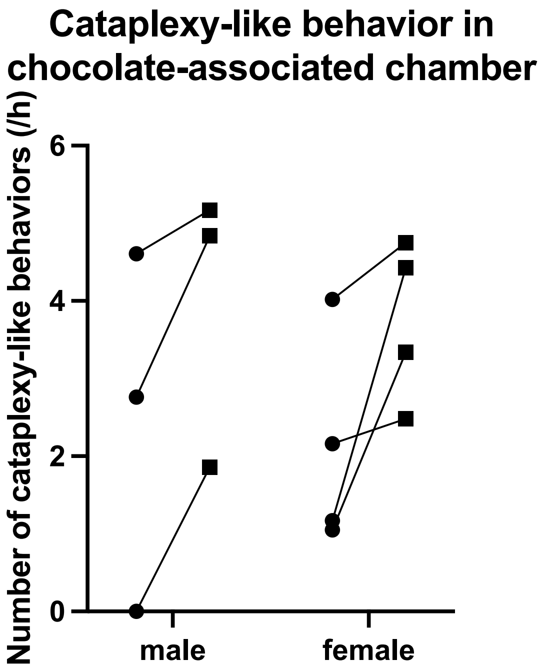

2way RM ANOVA results

Test day x gender	F(1,5) = 0.02768, P = 0.8744
Gender	F(1,5) = 0.05957, P = 0.8169
Test day	F(1,5) =12.21, P = 0.0174

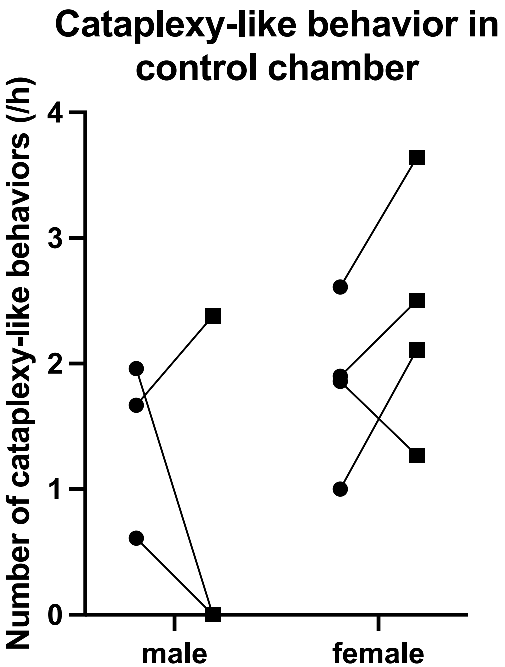

2way RM ANOVA results

Test day x gender	F(1,5) = 2.123, P = 0. 2049
Gender	F(1,5) = 2.768, P = 0.1570
Test day	F(1,5) =0.01078, P = 0.9213
